# Stereotactic body radiation therapy (SBRT) for prostate cancer in men with large prostates (≥50 cm^3^)

**DOI:** 10.1186/s13014-014-0241-3

**Published:** 2014-11-15

**Authors:** Einsley Janowski, Leonard N Chen, Joy S Kim, Siyuan Lei, Simeng Suy, Brian Collins, John Lynch, Anatoly Dritschilo, Sean Collins

**Affiliations:** Department of Radiation Medicine, Georgetown University Hospital, Washington DC, 20007 USA; Department of Urology, Georgetown University Hospital, Washington DC, 20007 USA

**Keywords:** Prostate cancer, Large prostate, SBRT, CyberKnife, Common toxicity criteria (CTC), Quality of life, EPIC, AUA, SF-12, Urinary symptom flare

## Abstract

**Background:**

Patients with large prostate volumes have been shown to have higher rates of genitourinary and gastrointestinal toxicities after conventional radiation therapy for prostate cancer. The efficacy and toxicity of stereotactic body radiation therapy (SBRT), which delivers fewer high-dose fractions of radiation treatment, is unknown for large prostate volume prostate cancer patients. We report our early experience using SBRT for localized prostate cancer in patients with large prostate volumes.

**Methods:**

57 patients with prostate volumes ≥50 cm^3^ prior to treatment with SBRT for localized prostate carcinoma and with a minimum follow up of two years were included in this retrospective review of prospectively collected data. Treatment was delivered using Cyberknife (Accuray) with doses of 35–36.25 Gy in 5 fractions. Biochemical control was assessed using the Phoenix definition. Toxicities were scored using the CTCAE v.4. Quality of life was assessed using the American Urological Association (AUA) Symptom Score and the Expanded Prostate Cancer Index Composite (EPIC)-26.

**Results:**

57 patients (23 low-, 25 intermediate- and 9 high-risk according to the D’Amico classification) at a median age of 69 years (range, 54–83 years) received SBRT with a median follow-up of 2.9 years. The median prostate size was 62.9 cm^3^ (range 50–138.7 cm^3^). 33.3% of patients received ADT. The median pre-treatment prostate-specific antigen (PSA) was 6.5 ng/ml and decreased to a median PSA of 0.4 ng/ml by 2 years (*p <*0.0001). A mean baseline AUA symptom score of 7.5 significantly increased to 13 at 1 month (*p = *0.001) and returned to baseline by 3 months (*p = *0.21). 23% of patients experienced a late transient urinary symptom flare in the first two years following treatment. Mean baseline EPIC bowel scores of 95.8 decreased to 78.1 at 1 month (*p* <0.0001), but subsequently improved to 93.5 three months (*p = *0.08). The 2-year actuarial incidence rates of GU and GI toxicity ≥ grade 2 were 49.1% and 1.8%, respectively. Two patients (3.5%) experienced grade 3 urinary toxicity, and no patient experienced grade 3 gastrointestinal toxicity.

**Conclusions:**

SBRT for clinically localized prostate cancer was well tolerated in men with large prostate volumes.

## Introduction

External beam radiation therapy (EBRT) and brachytherapy are the primary radiation modalities for clinically localized prostate cancer. Over the last few decades, external beam radiation delivery has evolved from 2-D delivery to 3-D conformal and, subsequently, to IMRT and stereotactic body radiation therapy (SBRT). These advances in radiation treatment planning and radiation delivery have allowed for higher doses of radiation to be delivered, thereby improving biochemical progression free survival [1-4] and reducing the rates of salvage therapy in high risk patients [5]. For men with localized prostate cancer, the typical treatment with dose-escalated EBRT involves fractionated radiation therapy, using daily doses of 1.8-2.0 Gy for eight to nine weeks. SBRT delivers fewer, high-dose fractions of radiation, providing a number of advantages, including a more convenient, shortened time course as well as a theoretical improvement in cancer response to larger daily radiation fractions [6]. Early data from trials of SBRT show SBRT to be comparably effective in prostate cancer treatment [7-15].

Brachytherapy, which involves placing radioactive sources into prostate tissue, is another radiation treatment option for patients who prefer an abbreviated course of treatment, either as a single modality in low risk patients or in combination with external beam therapy in intermediate and high risk patients [16,17]. However, brachytherapy is not appropriate for all patients, including patients with unfavorable anatomy [18], including pubic arch interference, those who cannot tolerate anesthesia, patients with significant medical co-morbidities, or those who are at increased risk for treatment-related complications. For instance, patients with a history of prior TURP treatment or with baseline urinary obstructive symptoms and/or large prostate sizes (>50 cc) are considered to be at an increased risk for urinary incontinence or urinary obstruction respectively. Indeed, the relationship between acute toxicity and large prostate size has been reported for brachytherapy [19-23], although one study has shown that biochemical control and acceptable late toxicities may be achieved in patients presenting with large volume prostates [24]. Some patients with large prostates may become candidates for brachytherapy with neoadjuvant androgen deprivation therapy (ADT) to shrink the prostate; however, this is achieved at the expense of toxicities of hormone therapy, including hot flashes, loss of libido, obesity, osteoporosis, and risk for diabetes and cardiovascular disease [25].

Acute toxicity associated with external beam radiation commonly involves the genitourinary and gastrointestinal systems, including irritative or obstructive urinary dysfunction and bowel frequency and urgency [26]. Advances in external beam radiation technique [27-29] and utilization of daily prostate localization with IGRT [30] have been shown to reduce the rates of toxicity. Like in brachytherapy, another factor that impacts the development of external beam radiation-induced toxicity is the volume of the prostate. The link between prostate volume and increasing acute radiation toxicity has been well described following 3-D conformal radiation [26,31] and IMRT [32].

While early data from SBRT shows a comparable toxicity profile to that of IMRT, the efficacy and toxicity of SBRT for prostate cancer patients with large prostate volumes remain unclear. The purpose of this study was to evaluate the influence of prostate volume on acute and late urinary and bowel toxicity in patients treated with SBRT.

## Methods

This study is a retrospective review of prospectively collected data on 57 consecutively treated patients with prostate volumes ≥50 cm^3^, who received hypofractionated stereotactic body radiotherapy at Medstar-Georgetown University Hospital as monotherapy for histologically-confirmed localized prostate cancer. Risk category was defined using the D’Amico classification [16]. Clinical stage was assigned according to the 6th edition of the American Joint Committee on Cancer definitions. Exclusion criteria included less than two years of clinical follow-up, clinical involvement of lymph nodes, distant metastases on pre-treatment imaging, prior prostate cancer-directed therapy, or prior pelvic irradiation. Institutional review board approval was obtained for this analysis.

### Treatment planning and delivery

**Figure 1:**
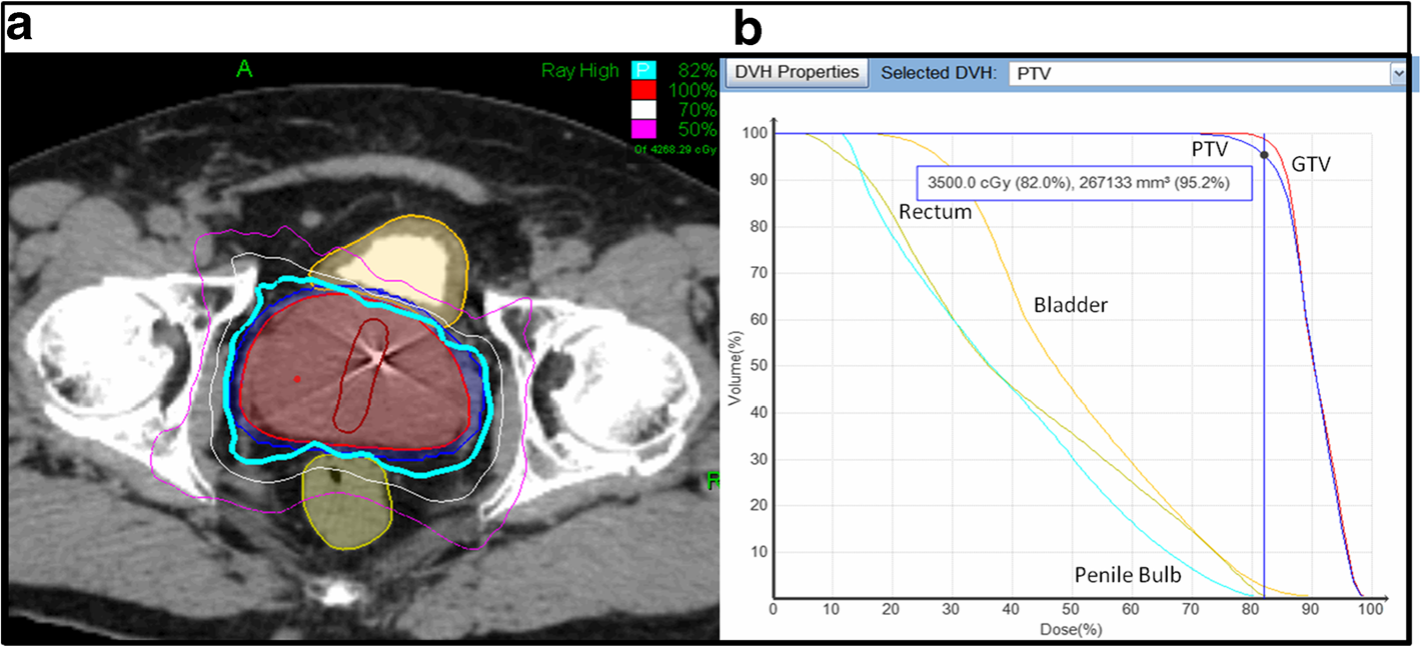
**Treatment Planning and Delivery. (a)** The volumes represent the GTV (red), PTV (blue), rectum (light green), and bladder (yellow). The prescription isodose line (82%) is denoted by the thick cyan line. **(b)** A typical dose-volume histogram (DVH) for Cyberknife treatment of a prostate cancer patient is shown, revealing doses to the GTV, PTV, and nearby critical structures, including the rectum, bladder, and penile bulb.

Prior to treatment planning, 4 gold fiducials were placed into the prostate. Approximately seven days after fiducial placement, patients underwent MR imaging followed shortly thereafter by a thin-cut CT scan. Fused CT and MR images were then used for treatment planning. The fiducial placement and CT/MRI simulation procedures have been previously described [33]. The gross tumor volume (GTV) was defined as the prostatic capsule and the proximal seminal vesicles up to the point that the left and right seminal vesicles separated. The GTV was then further expanded by 3 mm posteriorly and 5 mm in all other directions to create a CTV. No further expansion was made from CTV to PTV. Treatment planning was performed using Multiplan (Accuray Inc., Sunnyvale, CA). Figure 1a shows an axial view of a typical treatment plan. 35–36.25 Gy was given to the PTV in 5 fractions over 2 weeks to a mean prescription isodose line of 77%. The rectum, bladder, testes, penile bulb and membranous urethra and prostatic urethra were contoured and evaluated with a dose-volume histogram. A typical dose volume histogram is shown in Figure 1b. Dose constraints have been previously reported [7]. Target position was verified every 30–60 seconds during treatment using orthogonal kV images as previously described [33,34].

### Pretreatment assessment and follow-up

Prostate volume assessment was either performed by the patient’s treating urologist at the time of biopsy and/or at our institution during fiducial placement. In addition, patient assessments included clinical examination, a digital rectal exam, PSA level, and a quality of life (QOL) questionnaire. These assessments were performed prior to the initiation of stereotactic body radiotherapy, at 1 and 3 months post-treatment, and every 3 months thereafter. Biochemical failure was defined as a PSA rise of ≥2 ng/mL above the nadir [35]. Acute and late toxicities were scored using the Common Terminology Criteria for Adverse Events (CTCAE) Version 4.0. Toxicity is defined as acute, occurring within 6 months of completing treatment, and late reflecting those events occurring later than 6 months. At each follow-up visit, toxicity events were scored independently for each of the different toxicity types, and the highest GU and GI toxicities were determined for each patient. The QOL questionnaires evaluated urinary, bowel, and erectile functions using the American Urological Association Symptom Score (AUA) [36], the Expanded Prostate Cancer Index Composite (EPIC) short form [37], and the 12-item Medical Outcomes Study Short Form (SF-12) version 2 questionnaires [38]. Late urinary symptom flare was defined as having both an AUA score ≥15 with an increase of ≥5 points above baseline six months after the completion of SBRT.

### Statistical analysis

Student’s t-test and chi-square analysis were used to assess differences in ongoing PSA and quality of life scores in comparison to baseline. As previously reported, late urinary symptom flare was defined as an increase of ≥5 points above baseline with a degree of severity in the moderate to severe range (AUA score ≥15) [39]. The flare was considered resolved when either the AUA score dropped to <15 or the score returned to <5 points above the patient’s pre-treatment baseline. QOL data from time points in which more than 80% of patients completed the questionnaires were included in the analysis. A QOL change of one-half standard deviation (SD) from the baseline QOL score, defined as the minimal important difference (MID), was used to denote a clinically significant change in the QOL score [40]. The two-sided paired Wilcoxon rank-sum test was used to calculate the significance of differences in the mean scores on follow-up as compared to the baseline values. Parameters were identified as significant if the two-tailed p-value was less than 0*.*05. MedCalc® version 11.6.1.0 was used for the statistical analyses.

## Results

Fifty-seven men, who met the inclusion criteria, were treated at our institution between August 2008 and May 2011, with a median clinical follow-up of 2.9 years (range, 1.9-3.6 years). The median prostate size was 62.9 cm^3^ (range 50–138.7 cm^3^). Their baseline characteristics are summarized in Table 1. Of note, our patients were 54% Caucasian with a median age of 69 years old (range, 54–83 years). By D’Amico classification, 23 were low-, 25 intermediate-, and 9 high risk. 33.3% of our patients received androgen deprivation therapy as prescribed by their urologist for either combined modality prostate cancer treatment or management of urinary symptoms. 78.9% of patients were treated with 36.25 Gy and 19.3% of patients were treated with 35 Gy.

**Table 1:** **Patient characteristics**

**Age (yrs)**		**Percent patients (n = 57)**
	**<60**	**7**
	**60-69**	**45.6**
	**70-79**	**35.1**
	**≥80**	**12.3**
**Race**		
	**White**	**54.4**
	**Black**	**40.4**
	**Other**	**5.3**
**Pre-treatment PSA (ng/ml)**		
	**≤10**	**77.2**
	**10 < PSA ≤ 20**	**15.8**
	**>20**	**7**
**T-Stage**		
	**T1c**	**73.7**
	**T2a**	**12.3**
	**T2b**	**12.3**
	**T2c**	**1.8**
**Gleason score**		
	**5**	**1.8**
	**6**	**44**
	**7**	**42**
	**8**	**10.4**
	**Other**	**1.8**
**Risk group**		
	**Low risk**	**40.4**
	**Intermediate risk**	**43.9**
	**High risk**	**15.7**
**Hormone treatment**		
	**Yes**	**33**
	**No**	**67**
**Dose**		
	**35 Gy**	**19.3**
	**35.25 Gy**	**1.8**
	**36.25 Gy**	**78.9**
**α**_**1A**_ **Inhibitor (Flomax)**		
	**Yes**	**37**
	**No**	**63**

**Figure 2:**
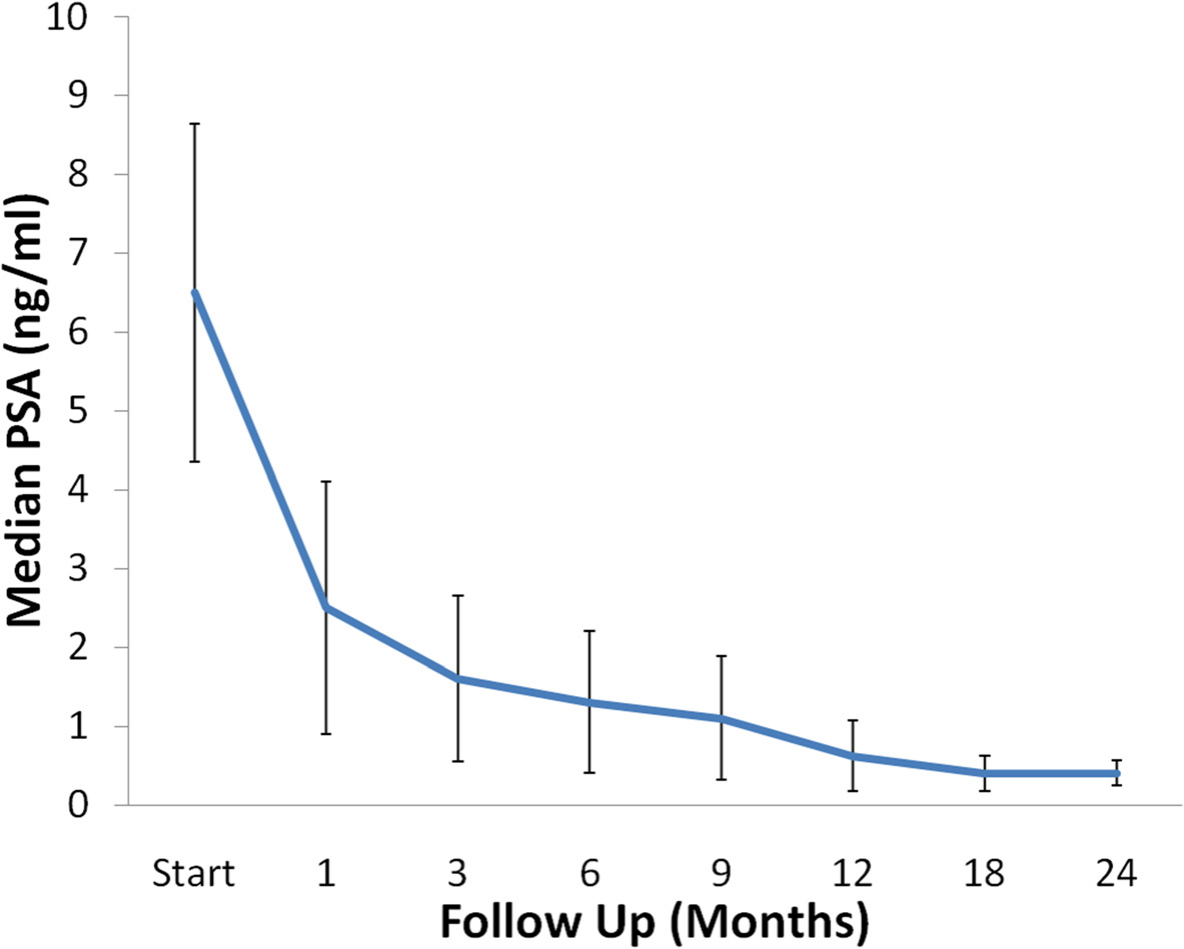
**Median PSA changes.** The median pre-treatment prostate-specific antigen (PSA) was 6.5 ng/ml, and, at 2 years, the median PSA decreased to 0.4 ng/ml (*p <* 0.0001).

The median pre-treatment PSA was 6.5 ng/mL (0.2-24 ng/mL), and, at 2 years, the median PSA decreased to 0.4 ng/mL (0.1-1.7 ng/mL) (*p* <0.0001) (Figure 2). There was one biochemical failure, occurring in an intermediate risk patient, who was found to have widely metastatic disease to the bone 12 months post-treatment. The overall two-year actuarial biochemical relapse free survival was 98%.

Baseline quality of life demographics for our patients are shown in Table 2. Pre-treatment EPIC results reveal that our patient population had baseline mild-moderate urinary incontinence (score 86.4) and irritative/obstructive (score 86) symptoms. The low sexual function scores may be partially related to the 33.3% use of androgen deprivation therapy. Pre-treatment AUA scores correlate with the findings on EPIC, revealing that the majority of our treatment population had either mild (49.1%) or moderate (43.9%) baseline urinary bother, with the mean AUA score being 9.1 ± 6.6 (range, 0–33). Baseline SF-12 scores for our patient group were comparable to those of a similarly aged general population.

**Table 2:** **Baseline QOL characteristics**

**EPIC Individual Domain Score (mean)**		
	**Urinary Incontinence**	**86.4**
	**Urinary Irritative/Obstructive**	**86**
	**Bowel**	**95.8**
	**Sexual**	**41.3**
	**Hormonal**	**88.2**
**Baseline SF-12 Score**		
	**PCS (n = 56)**	**48.9 (22.1-63.1)**
	**MCS (n = 56)**	**56.7 (31.1-65.9)**
**Baseline AUA Score**		**Patient No. (% patients)**
	**0-7 (mild)**	**28 (49.1%)**
	**8-19 (moderate)**	**25 (43.9%)**
	**≥20**	**3 (5.3%)**

**Figure 3:**
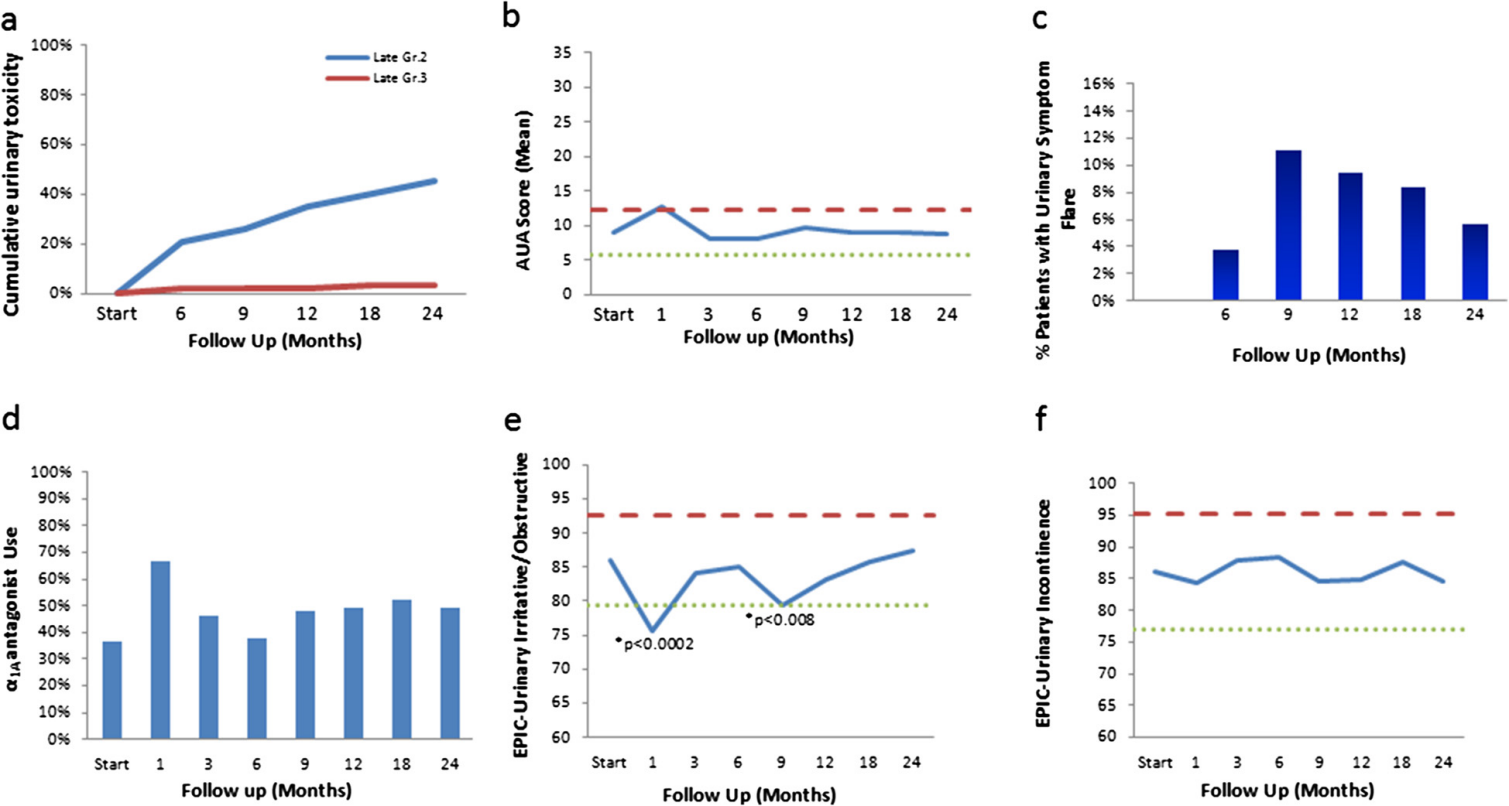
**Urinary Toxicity and Quality of Life. a)** Cumulative late urinary toxicity (grade 2 in blue and grade 3 in red). **b)** Average AUA score. AUA scores range from 0–35 with higher values representing worsening urinary symptoms. **c)** Percent of patients with urinary symptom flare, which was defined as having both an AUA score ≥15 with an incrase of ≥5 points above baseline. **d)** Percent of patients using α-antagonists at baseline and at different time points at follow up. **e-f)** Average EPIC urinary irritative/obstructive **(e)** and incontinence **(f)** scores. The thresholds for clinically significant changes in scores (1/2 standard deviation above and below the baseline) are indicated by the red and green lines. EPIC score range from 0–100 with higher values representing a more favorable health-related quality of life.

The prevalence of GU and GI toxicities following treatment is shown in Table 3. The prevalence of single symptoms as well as the highest GI and GU toxicity per patient are depicted independently for each follow-up visit. Actuarial incidence rates of late grade 2 and 3 GU toxicities are shown in Figure 3a. 49.1% of patients experienced grade 2 urinary toxicities, requiring initiation or increase in alpha antagonist medications. Urinary grade 3 toxicities were low, being observed in 3.5% of patients, with one patient developing grade 3 retention at 12 months requiring a TURP and another patient developing grade 3 hematuria at 6 months requiring cauterization.

**Table 3:** **Prevalence of CTC graded GI and GU toxicities**

**Toxicity**	**Grade**	**Start**	**1**	**3**	**6**	**9**	**12**	**18**	**24**
**Diarrhea**	**0**	**100%**	**53%**	**84%**	**75%**	**83%**	**88%**	**94%**	**94%**
	**1**	**0%**	**35%**	**16%**	**23%**	**15%**	**12%**	**6%**	**6%**
	**2**	**0%**	**12%**	**0%**	**2%**	**2%**	**0%**	**0%**	**0%**
**Proctitis**	**0**	**86%**	**79%**	**93%**	**92%**	**100%**	**98%**	**100%**	**100%**
	**1**	**14%**	**21%**	**7%**	**8%**	**0%**	**2%**	**0%**	**0%**
	**2**	**0%**	**0%**	**0%**	**0%**	**0%**	**0%**	**0%**	**0%**
**Rectal Bleeding**	**0**	**100%**	**91%**	**95%**	**100%**	**98%**	**98%**	**96%**	**96%**
	**1**	**0%**	**9%**	**5%**	**0%**	**2%**	**2%**	**4%**	**4%**
	**2**	**0%**	**0%**	**0%**	**0%**	**0%**	**0%**	**0%**	**0%**
**Highest GI**	**0**	**67%**	**46%**	**77%**	**75%**	**82%**	**86%**	**91%**	**91%**
	**1**	**33%**	**42%**	**23%**	**23%**	**16%**	**14%**	**9%**	**9%**
	**2**	**0%**	**12%**	**0%**	**2%**	**2%**	**0%**	**0%**	**0%**
**Hematuria**	**0**	**98%**	**95%**	**93%**	**94%**	**91%**	**90%**	**91%**	**94%**
	**1**	**2%**	**5%**	**4%**	**2%**	**4%**	**4%**	**7%**	**2%**
	**2**	**0%**	**0%**	**4%**	**2%**	**4%**	**4%**	**0%**	**2%**
	**3**	**0%**	**0%**	**0%**	**2%**	**2%**	**2%**	**2%**	**2%**
**Dysuria**	**0**	**89%**	**65%**	**77%**	**90%**	**80%**	**92%**	**91%**	**92%**
	**1**	**11%**	**35%**	**23%**	**10%**	**20%**	**8%**	**9%**	**8%**
	**2**	**0%**	**0%**	**0%**	**0%**	**0%**	**0%**	**0%**	**0%**
**Incontinence**	**0**	**75%**	**67%**	**71%**	**79%**	**74%**	**77%**	**76%**	**73%**
	**1**	**21%**	**30%**	**27%**	**17%**	**24%**	**21%**	**19%**	**27%**
	**2**	**4%**	**4%**	**2%**	**4%**	**2%**	**2%**	**6%**	**0%**
**Urinary frequency/ urgency**	**0**	**39%**	**16%**	**55%**	**62%**	**48%**	**52%**	**48%**	**58%**
	**1**	**61%**	**82%**	**45%**	**35%**	**50%**	**44%**	**48%**	**42%**
	**2**	**0%**	**2%**	**0%**	**4%**	**2%**	**4%**	**4%**	**0%**
**Retention**	**0**	**67%**	**33%**	**57%**	**54%**	**57%**	**56%**	**54%**	**44%**
	**1**	**33%**	**26%**	**20%**	**21%**	**19%**	**15%**	**19%**	**29%**
	**2**	**0%**	**53%**	**29%**	**30%**	**29%**	**31%**	**31%**	**36%**
	**3**	**0%**	**0%**	**0%**	**0%**	**0%**	**2%**	**2%**	**2%**
**Highest GU**	**0**	**33%**	**5%**	**25%**	**32%**	**26%**	**32%**	**32%**	**37%**
	**1**	**63%**	**53%**	**51%**	**46%**	**49%**	**42%**	**39%**	**35%**
	**2**	**10%**	**89%**	**50%**	**40%**	**46%**	**42%**	**44%**	**39%**
	**3**	**0%**	**0%**	**0%**	**2%**	**2%**	**2%**	**4%**	**4%**

Analyses of urinary function health related quality of life are shown in Figures 3b-f. Evaluation of AUA score (Figure 3b) reveals two distinct peaks at 1 month and 9 months post-treatment. A mean baseline AUA symptom score of 7.5 significantly increased to 13 at 1 month (*p* = 0.001) and returned to baseline at 3 months (*p* = 0.21). Another small peak was seen between 6 to 9 months, where the mean AUA went from 9 to 9.7. Indeed, 23% of patients experienced a late transient urinary symptom flare (≥6 months after completing treatment) in the first two years following treatment (Figure 3c), with the median time to flare being 9 months and the median duration of flare being 3 months. As shown in Figure 3d, at baseline, 37% of patients reported using alpha antagonists pre-treatment. New or increased alpha antagonist use, graded as a grade 2 toxicity (Table 3), correlated with the increased AUA score and peaked at 1 month, with 67% of patients requiring change or initiation of alpha antagonist medications. Another small increase in alpha antagonist use was seen from 6 to 9 months post-treatment, also correlating with the second increased AUA score. At 24 months alpha antagonist use was higher than baseline but significantly decreased from the peak of 1 month, with 48% of patients reporting use at two years. Analysis of the changes in EPIC scores (Figures 3e-f) also showed two significant dips at 1 month and at 9 months. As shown in Figure 3e, mean baseline EPIC urinary obstructive scores of 86 significantly decreased to 75.5 at one month (*p* <0.0002), but subsequently improved to 84 at 3 months (*p* = 0.39). Another transient decrease in EPIC scores occurred at 9 months, with the 6 month score of 85 decreasing to 90 at 9 months (*p* <0.008). EPIC urinary obstructive scores subsequently improved back to baseline at 12 months (*p* = 0.13) and remained stable, with the 2 year score being 87.4. The mean baseline EPIC urinary incontinence score of 86.1 (Figure 3f) was not significantly changed throughout the 2 year post-treatment follow up.

**Figure 4:**
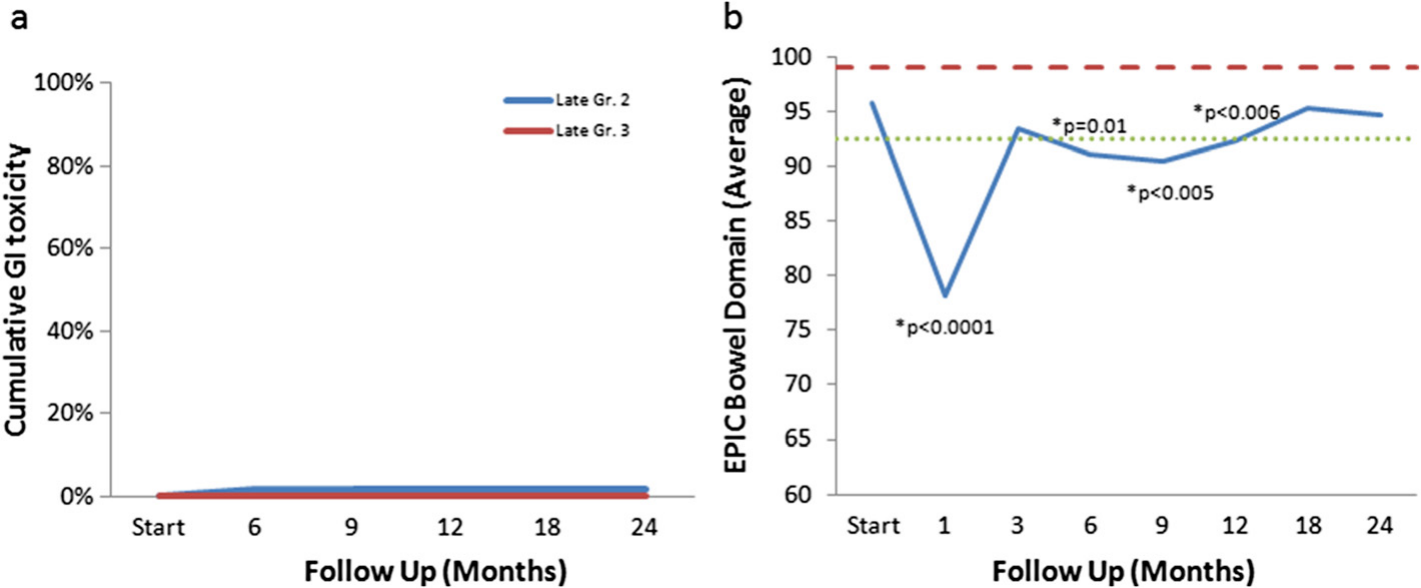
**Gastrointestinal Toxicity and Quality of Life. a)** Cumulative late gastrointestinal toxicity (grade 2 in blue and grade 3 in red). **b)** Average EPIC bowel domain scores. The thresholds for clinically significant changes in scores (1/2 standard deviation above and below the baseline) are indicated by the red and green lines. EPIC score range from 0–100 with higher values representing a more favorable health-related quality of life.

Actuarial incidence rates of late grade 2 and 3 GI toxicities are shown in Figure 4a. A grade 2 GI toxicity of increased frequency of bowel movements was seen in one patient (1.8%); there were no grade 3 gastrointestinal toxicities. The bowel quality of life scores are shown in Figure 4b. The mean EPIC bowel score of 95.8 significantly decreased to 78.1 at 1 month (*p* <0.0001), but subsequently improved to 93.5 at three months (*p* = 0.08). Another much smaller decline was seen at 9 months (*p* <0.005), but improved at 12 months and remained steady, with a score of 94.7 at two years (*p* = 0.32).

## Discussion

A variety of treatment modalities for prostate cancer are available to patients, with retrospective series showing comparable biochemical local control rates for low-risk patients treated with surgery, brachytherapy, and external beam radiation [41]. However, not all treatment modalities are appropriate for all patients, and treatment recommendation decisions are often stratified by patient convenience and preference, toxicity-related quality of life, and cancer risk category. Although IMRT is the standard external beam modality to treat clinically localized prostate cancer, disadvantages include a treatment course of 8–9 weeks. SBRT delivers highly conformal, high dose radiation in 5 or fewer treatment fractions, providing convenience to the patients and a theoretical improvement in cancer response to larger daily radiation fractions [6].

A growing body of evidence has established the efficacy of SBRT for prostate cancer treatment, with multiple single institution studies of SBRT reporting a biochemical relapse free survival of 90-100% with a median follow up of 5 years or more [8,10,12,42-45]. In this SBRT study, our two year biochemical relapse free survival of a diverse group of low-, intermediate-, and high risk patients is consistent with that of other institutions.

Treatment of prostate cancer patients with large prostates can provide new challenges. Indeed, radiation treatment of such patients is associated with increased acute urinary toxicity compared to patients presenting with small prostates. NCCN guidelines currently recommend caution for brachytherapy treatment of patients with large prostates based on widely published data relating the increased frequency of acute urinary obstructive and irritative symptoms requiring catherization or other interventions [19,21,22,26,46,47].

The relationship between prostate volume and external beam radiation related toxicity has not been as widely documented, but the correlation between increasing urinary toxicity and increasing prostate volume also appears to hold true for 3D conformal radiation and IMRT [31,32]. Pinkawa *et al.* prospectively assessed health related quality of life in patients with prostate volumes stratified by prostate size less than or greater than or equal to 44 cm^3^, finding that patients with large prostates had significantly worse urinary bother scores immediately after radiation, but that the two groups were not different at 2 and 16 months after treatment [31]. Aizer *et al.* retrospectively reviewed the genitourinary toxicity of patients treated with IMRT stratified by prostate size into less than or greater than 50 cm^3^, demonstrating the patients with large prostate volumes had significantly higher acute urinary frequency/urgency and grade 3 GU toxicity [32]. There was no difference between the two groups in urinary retention or incontinence.

In addition to the convenience of the timing of treatment, intra-fraction image guidance during treatment allows for smaller CTV-PTV margins, thereby minimizing dose to nearby critical structures to reduce treatment related toxicity. SBRT treatment of localized prostate cancer in patients with large prostates was well tolerated by patients at our institution. Our largely grade 1–2 toxicities were symptomatically managed. If initiation or increase of alpha antagonists was not sufficient to manage symptoms, a short 1 week course of steroids were given with symptomatic improvement, as previously reported [48]. Grade 3 urinary toxicities were infrequent in our SBRT patients, occurring less commonly than predicted by the prior large prostate radiation studies using different radiation modalities [31,32,49]. When compared to our previous publication of prostate cancer patients treated with SBRT not stratified by prostate volume, it appears that patients with large prostate volumes do have a higher rate of grade 2 or higher toxicity [7]. Our results are consistent with those of Pinkawa and Aizer regarding the trend towards increased acute irritative obstructive symptoms and toxicity in patients with large prostates treated with external beam radiation.

Late urinary symptom flares have been commonly observed in prostate cancer patients treated with radiation. This was first observed by Cesaretti *et al.* in 2003 as occurring a median of 23.9 months after brachytherapy [50]. Keyes *et al.* showed that a greater baseline International Prostate Symptom Score was positively correlated with the development of the late urinary symptom flare [49]. This late urinary symptom flare has also been described in SBRT [7,9]. While the etiology of the flare is unknown, theories include late radiation damage to the smooth muscle or vasculature of the prostate, causing late radiation induced prostatitis/urethritis. In our patients, these late urinary symptom flares were observed, and the majority resolved with conservative management.

Grade 2 or higher GI toxicities were rare in our patients. Assessment of quality of life showed an acute worsening of EPIC bowel scores at one month post-treatment. These findings are comparable to those of conventionally fractionated radiation therapy [26,51,52], and the symptoms were managed conservatively with either observation or medications. To our knowledge, this is the first study to report a late GI symptom flare with SBRT. Interestingly, a link between late urinary symptom flare and rectal toxicity in brachytherapy patients has been previously described and may relate to a patient’s ability to repair radiation-induced DNA damage [49].

## Conclusions

We report here, the first study on toxicity and health related quality of life in patients presenting with large prostate volumes treated with SBRT. This report supports the conclusion that patients with larger prostate volumes can be safely treated with SBRT, and gastrointestinal and urinary toxicity rates are comparable, if not improved, to those observed in conventionally fractionated radiation therapy and brachytherapy. Based on continued reports on the safety and efficacy of SBRT as well as patient preference for a shorter treatment course, SBRT utilization is likely to continue to increase. Our institutional experience adds to the growing body of evidence supporting the effectiveness and safety of SBRT even for larger prostate volumes.

## Consent

Written informed consent was obtained from patients for the publication of this report and any accompanying images.

## Abbreviations

ADT: Androgen deprivation therapy
AUA: American Urological Association
CT: Computed tomography
CTCAE: Common Terminology Criteria for Adverse Events
CTV: Clinical target volume
DVH: Dose-volume histogram
EBRT: External beam radiation therapy
EPIC: Expanded prostate index composite
GI: Gastro-Intestinal
GTV: Gross target volume
GU: Genito-Urinary
IGRT: Image-Guided Radiation Therapy
IMRT: Intensity Modulated Radiation Therapy
MRI: Magnetic Resonance Imaging
PTV: Planning target volume
QoL: Quality of life
SBRT: Stereotactic body radiation therapy
